# Mass screening for liver cancer: results from a demonstration screening project in Zhongshan City, China

**DOI:** 10.1038/s41598-018-31119-9

**Published:** 2018-08-24

**Authors:** Mingfang Ji, Zhiwei Liu, Ellen T. Chang, Xia Yu, Biaohua Wu, Li Deng, Qianjin Feng, Kuangrong Wei, Xuejun Liang, Shifeng Lian, Wen Quan, Panpan Wang, Yun Du, Zhiheng Liang, Shenglin Xia, Hai Lin, Fugui Li, Weimin Cheng, Weiqiang Chen, Yong Yuan, Weimin Ye

**Affiliations:** 1 Cancer Research Institute of Zhongshan City, Zhongshan City People’s Hospital, Zhongshan, Guangdong, P. R. China; 2 Department of Medical Epidemiology and Biostatistics, Karolinska Institutet, Stockholm, Sweden; 3 Division of Cancer Epidemiology and Genetics, , National Cancer Institute, 9609 Medical Center Drive, Rockville, Maryland USA; 4 Exponent, Inc., Health Sciences, Menlo Park, California, USA; 5 Division of Epidemiology, Department of Health Research and Policy, Stanford University School of Medicine, Stanford, California, USA; 6 Institute for Prevention & Control of Non-Communicable Chronic Diseases, Zhongshan Center for Disease Control and Prevention, Zhongshan, Guangdong, P. R. China; 7 Sun Yat-sen University Cancer Center, State Key Laboratory of Oncology in South China, Collaborative Innovation Center for Cancer Medicine, Guangzhou, P. R. China

## Abstract

Current Chinese national guidelines recommend routine screening for liver cancer in patients positive for HBsAg, irrespective of fibrosis status, age, or family history of liver cancer. We aim to evaluate whether the recommended screening strategy could reduce liver-cancer-specific mortality. We conducted a liver cancer mass screening trial in Xiaolan Town, Zhongshan City, China, among residents aged 35–64 years in 2012. All volunteers were offered serological testing for hepatitis B virus surface antigen (HBsAg). We proposed biannual screening using serum alpha-fetoprotein (AFP) and ultrasonography examination for subjects positive for HBsAg. Among 17,966 participants (26.2% of 68,510 eligible residents) who were free of liver cancer at baseline in 2012, we identified 57 incident cases of liver cancer within the first 4 years of follow-up (i.e., 43 among 2,848 HBsAg-positive participants and 14 among 15,118 HBsAg-negative participants), compared with 104 cases identified in non-participants (N = 50,544). A total of 207 participants had the recommended number of ultrasonography examinations (every 6 months) during the screening period. Compared with cases identified from non-participants, the cases arising among participants were more likely to be at early stage and had better survival than those among non-participants. However, we did not observe a reduction in liver cancer-specific mortality rate among participants (relative risk = 1.04, 95% confidence interval = 0.68, 1.58, *P* = 0.856). Our demonstration screening study does not show a reduction in liver cancer mortality within the first 4 years of follow-up according to current guidance in China, although long-term efficacy remains to be evaluated. Targeted surveillance among high-risk individuals as recommended by international guidelines, along with measures to improve compliance, should be evaluated in the Chinese population.

## Introduction

Liver cancer is the second leading cause of cancer death worldwide, with ~746,000 deaths in 2012^1,2^. Of ~782,000 new liver cancer cases diagnosed every year, more than half occur in China^1–3^. Chronic infection with hepatitis B virus (HBV) is the most common etiological factor for liver cancer in China^4,5^. Since 2002, universal newborn HBV vaccination has been instituted, which is expected to lead to future reductions in liver cancer^6^. However, individuals born before initiation of the vaccination program are not protected by HBV immunization (although many are protected naturally by antibody-mediated immunity following resolution of HBV infection). The prognosis of liver cancer patients is generally poor, with 5-year survival rate less than 5%; early-stage liver cancer, however, is amenable to curative therapy, enabling 5-year survival rate of 70% or higher^7,8^. Such evidence highlights the potential of secondary prevention, with a focus on screening and early detection, to reduce liver cancer mortality in populations not covered by the newborn vaccination program.

Although screening for liver cancer can enable early diagnosis of primary liver cancer, whether systematic screening reduces liver cancer mortality is uncertain. In China, the current recommended screening approach is testing for HBV surface antigen (HBsAg), which indicates chronic HBV infection, followed by a combination of serum alpha-fetoprotein (AFP) measurement and ultrasonography in those who are HBsAg-positive^9^. This recommendation is different from the current American Association for the Study of Liver Disease (AASLD) and Asian Pacific Association for the Study of the Liver (APASL) guidelines, which focusing on screening among non-cirrhotic HBsAg-positive patients ages >40 years for males, >50 years for females, or any age for those with a positive family history of liver cancer^10,11^. However, only two randomized controlled screening trials for liver cancer have been reported from China, with conflicting results^12,13^. Zhang *et al*.^13^ reported that semiannual screening with AFP and liver ultrasonography in those with HBV infection or chronic hepatitis significantly reduced liver cancer mortality by 37% in urban Shanghai. By contrast, a trial of semiannual AFP screening among HBsAg-positive individuals in Qidong failed to detect a significant reduction in mortality^12^, due partly to ineffective therapy for liver cancer patients found by screening. Interpretation of this result was hampered by the lack of published information on randomization technique and allocation concealment, and the sparsely reported results on baseline characteristics and liver cancer incidence in the intervention and control groups^14,15^. These limitations preclude a firm conclusion that screening with AFP and ultrasonography examination in an HBsAg-positive population can reduce liver cancer mortality.

Better understanding of the strategy for screening for liver cancer, including the frequency and management of positive screening results, can inform development and implementation of effective screening programs for liver cancer. Therefore, in 2012, we conducted a demonstration project of liver cancer mass screening in Xiaolan Town, a community in southern China with an intermediate incidence of liver cancer relative to other areas in China^16,17^. The aim of this project is to evaluate whether the currently recommended screening strategy in China, applied to the general population at the community level rather than targeting a subset of high-risk HBsAg-positive patients, could reduce liver-cancer-specific mortality. In this report, we present results on screening for primary liver cancer, diagnosis, and prognosis after an average of 4 years of follow-up.

## Methods

### Study Population

**Figure 1:**
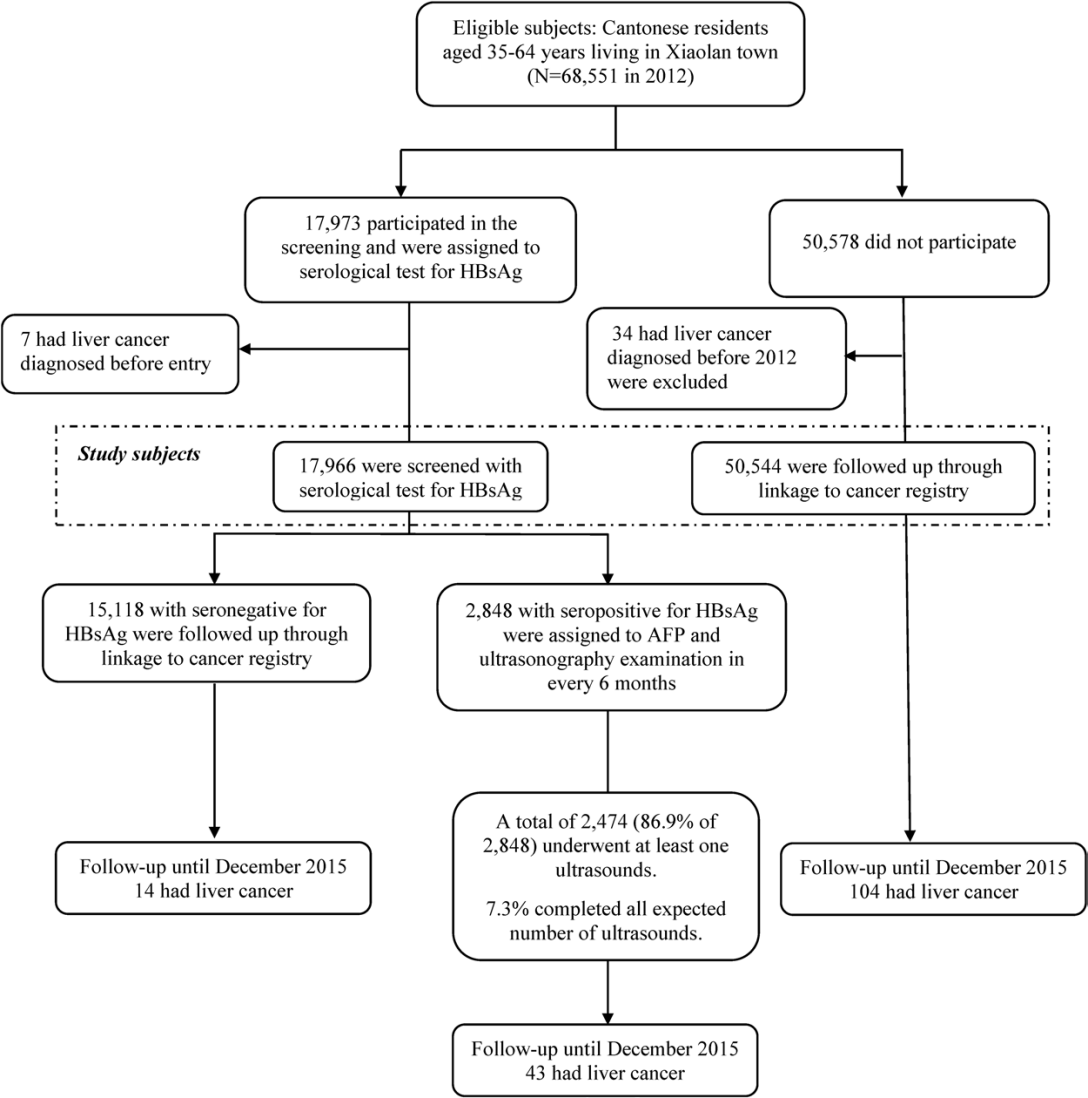
Enrollment and follow-up of the study participants after the initial screening in Xiaolan town (community), Zhongshan City, China.

A community-based mass screening trial was initiated among residents aged 35–64 years in Xiaolan Town, Zhongshan City, China, in 2012 (N = 68,551, Fig. 1). Selection of the target community was based on several practical considerations, including the high completeness of the local cancer registration system, high quality of the health care practitioners, and low residential mobility of the population. To maximize the participation rate, residents of the target community were contacted by their residential healthcare providers and government officials, who introduced the screening program with information leaflets containing general information on liver cancer, the potential benefits and risks of the screening program, and the planned follow-up strategies in case of a positive screening test result. This study (NCT02501980, registered at ClinicalTrials.gov) was approved by the Ethics Review Committee of the Zhongshan City People’s Hospital (ZSKY2012 [02]). All methods were performed in accordance with the relevant guidelines and regulations. Written informed consent was obtained from all participants.

### Screening test

All residents who voluntarily participated in the screening trial were asked to provide 8 mL blood collected in 2 tubes, 1 with EDTA-K2 and 1 without. All blood samples were processed within 8 hours at local hospital laboratories, where samples were separated into serum, plasma, and buffy coat and stored at −20 °C until being tested. Serum samples were tested for HBsAg by ELISA (Autobio Diagnostics Co., China). Samples with optical density/reference ≥1.0 were classified as positive. All positive samples were additionally tested by immunogum gold paper assay; the positive agreement was 98.7% between the two assays. Samples that were positive for both assays were classified as HBsAg seropositive. Serum samples from HBsAg-positive participants were further tested for AFP by ELISA (Autobio Diagnostics Co.). Samples positive for AFP and a subset of AFP-negative samples (1%) were additionally tested by another commercial ELISA kit (Wantai Biopharm, China); the agreement was 100% between the two assays. Liver ultrasonography was also conducted among participants who were HBsAg positive regardless of AFP levels. Participants with a high AFP level (≥20 ng/mL) and/or an abdominal mass detected by ultrasonography, were invited for a computerized tomography (CT) scan or magnetic resonance imaging (MRI) of the liver in the regional hospital (Xiaolan Town People’s Hospital). Patients with evidence of liver cancer based on imaging were immediately given advice for treatment.

### Screening interval and follow-up

Figure 1 shows recruitment and evaluation of the screening trial. Briefly, participants positive for HBsAg at study entry in 2012 but without a diagnosis of liver cancer (regardless of AFP and liver ultrasonography results) were rescreened every 6 months. Residents of Xiaolan Town were also followed for cancer incidence, vital status, and relocation by linkage to the Zhongshan Cancer Register and town-level death and migration registers. The Zhongshan Cancer Register was established in 1970 and collects information from all resources of Zhongshan, including the cancer research institute, regional hospitals in each town or community, and community health service stations. In addition, if cancer patients are diagnosed and/or treated in hospitals outside Zhongshan, information is collected annually. Primary liver cancer cases among HBsAg-positive participants were identified by the research team using CT scan or MRI according to the study protocol, and by annual linkage to the Zhongshan Cancer Register and Death Register. Liver cancer cases among non-participants and HBsAg-negative participants were identified only through linkage to the Cancer Register and Death Register. We reviewed all medical records of the identified liver cancer cases to verify the diagnosis and underlying causes of death (for those deceased), and collected detailed clinical data (e.g., pathological diagnosis, imaging records, tumor stage). For cases identified from the Death Register but not recorded in the Cancer Register, we also reviewed their medical records to confirm the diagnosis. For those deceased but without underlying causes of death recorded in medical files, we assessed such information through Death Register or through interviews of the local health care providers.

### Statistical analysis

The primary analysis compared liver cancer mortality between participants and non-participants. Person-years at risk were counted from enrollment (January 2012 to November 2012, for participants, or January 1, 2012, for non-participants) until death, emigration out of Zhongshan City, or December 31, 2015, whichever occurred first. We also conducted secondary analyses comparing liver cancer incidence rate between the two groups, where person-years at risk were counted from enrollment (or January 1, 2012, for non-participants) until liver cancer diagnosis, death, emigration out of Zhongshan, or December 31, 2015, whichever occurred first. We used log-linear Poisson regression models to calculate relative risks (RRs) and 95% confidence intervals (CIs), adjusted for age and sex, for all-cause or liver-cancer-specific mortality or liver cancer incidence comparing participants with non-participants. Disease-specific cumulative incidence and mortality curves were plotted by using the Nelson-Aalen estimate of the cumulative hazard function. Overall survival curves were estimated using the Kaplan-Meier method for liver cancer cases identified from the participants and non-participants, and compared using the log-rank test. We used Cox proportional hazards regression models to estimate hazard ratios (HRs) and 95% confidence intervals (CIs) with overall survival, adjusted for age and sex.

We used several approaches to account for potential confounding by sex and age due to the nonrandomized nature of this study. First, we conducted stratified analyses by sex and age groups. Second, to determine whether liver cancer incidence in the target community was similar to that of the general population in Zhongshan City after adjusting for sex and age, we calculated the standardized incidence ratio (SIR) of liver cancer among residents compared with the general population of Zhongshan City in 2012–2013. We also estimated the sex- and age-standardized mortality ratio (SMR) for all causes of death in the target community compared with the general population of Zhongshan City in 2012–2013. Finally, to investigate whether the observed incidence difference was due to difference in HBsAg prevalence among participants compared with non-participants, we tested HBsAg among 500 subjects randomly selected from those who attended a screening trial for nasopharyngeal carcinoma but did not attend the screening trial for liver cancer, frequency matched to the distribution of age and sex of the participants in the screening trial for liver cancer. Because cases detected within one year of follow-up could be the prevalent cases thus might not benefit from screening, we reanalyzed the data by starting follow-up one year after the date of enrollment.

Analyses were conducted using SAS version 9.4 software (SAS Institute, Inc., Cary, North Carolina). All statistical tests were two-sided and a *P* value of <0.05 was considered statistically significant. All authors had access to the study data and had reviewed and approved the final manuscript.

### Power calculation

With a total of 17,973 participants (experimental group) and 50,578 non-participants (control group), this study was designed with 80% power to detect a liver-cancer mortality RR of 0.87 or less (more extreme) after 4 years of follow-up.

## Results

### Characteristics of the residents and use of screening

A total of 17,973 (26.2% of 68,551) eligible residents of Xiaolan Town aged 35–64 years voluntarily participated in the screening project (Fig. 1). We excluded 7 cases of primary liver cancer diagnosed before recruitment, leaving 17,966 participants. Among 50,578 non-participants, 34 individuals diagnosed with liver cancer before 2012 were also excluded, leaving 50,544 non-participants for analysis.

Table 1 shows the demographic characteristics in the participant and non-participant groups. Participants were more likely to be male and were slightly older than non-participants. All HBsAg seropositive participants (N = 2,848) underwent baseline serum AFP testing, of whom 61.8% (1760 of 2848) participated baseline liver ultrasonography examination. Due to a long preparation period (~9 months), the first round of AFP/ultrasonography examination was conducted between October 2012 and August 2013. The subsequent follow-up tests were conducted in April 2014, October 2014, April 2015 and October 2015, respectively, totaling 5 times of follow-up tests (Table 2 and Supplementary Table 1). A total of 2,474 (86.9% of 2,848) underwent at least one ultrasonography examination and 2,023 (71.0% of 2,848) underwent at least two during the screening period. A total of 207 (7.3% of 2,848 HBV carriers, 183 non-cases and 24 cases completed all expected number of ultrasounds) participants had the expected number of ultrasounds (every 6 months) during the screening period. The median time between 2 ultrasounds was 10 months (range: 4–43 months).

**Table 1:** Baseline characteristics of residents in Xiaolan Town (Community) by intervention group, Zhongshan City, Guangdong Province, China, 2012–2015.

Variable	Participants (N = 17,966)		Non-participants (N = 50,544)		*P* value*
	No. of subjects	%	No. of subjects	%	
Sex					<0.001
Male	10,555	58.8	21,603	42.7	
Female	7,411	41.2	28,941	57.3	
Age in 2012 (years)					<0.001
35–39	1,820	10.1	9,624	19.0	
40–49	6,489	36.1	20,550	40.7	
50–59	6,752	37.6	14,313	28.3	
60–64	2,905	16.2	6,057	12.0	
First-degree family history of cancer					—
No	16,943	94.3	—	—	
Cancers other than liver	754	4.2	—	—	
Liver cancer	269	1.5	—	—	

*P value from Chi-squared test.

**Table 2:** Number of ultrasonography examinations during baseline and follow-up among subjects positive for hepatitis B surface antigen (N = 2848), 2012–2015*.

Total (%)		Gender		*P* value	Age in 2012 (years)				*P* value
		male (n = 1,914)	female (n = 934)		35–39 (n = 421)	40–49 (n = 1,173)	50–59 (n = 974)	60–64 (n = 280)	
Number of ultrasonography examinations				0.411					<0.001
0	374 (13.1)	246 (12.8)	128 (13.7)		81 (19.2)	182 (15.5)	86 (8.8)	25 (8.9)	
1	451 (15.8)	300 (15.7)	151 (16.2)		73 (17.3)	184 (15.7)	156 (16.0)	38 (13.6)	
2	390 (13.7)	278 (14.5)	112 (12.0)		55 (13.5)	178 (15.2)	118 (12.1)	39 (13.9)	
3	440 (15.4)	295 (15.4)	145 (15.5)		57 (13.5)	158 (13.5)	163 (16.7)	62 (22.1)	
4	530 (18.6)	352 (18.4)	178 (19.1)		66 (15.7)	210 (17.9)	195 (20.0)	59 (21.1)	
5	478 (16.8)	328 (17.1)	150 (16.1)		69 (16.4)	186 (15.9)	180 (18.5)	43 (15.4)	
6	185 (6.5)	115 (6.0)	70 (7.5)		20 (4.7)	75 (6.4)	76 (7.8)	14 (5.0)	

*Dates for the first to the fifth round of re-examinations: October 2012 - August 2013, April 2014, October 2014, April 2015 and October 2015, respectively.

### Liver cancer incidence and mortality among participants and non-participants

**Figure 2:**
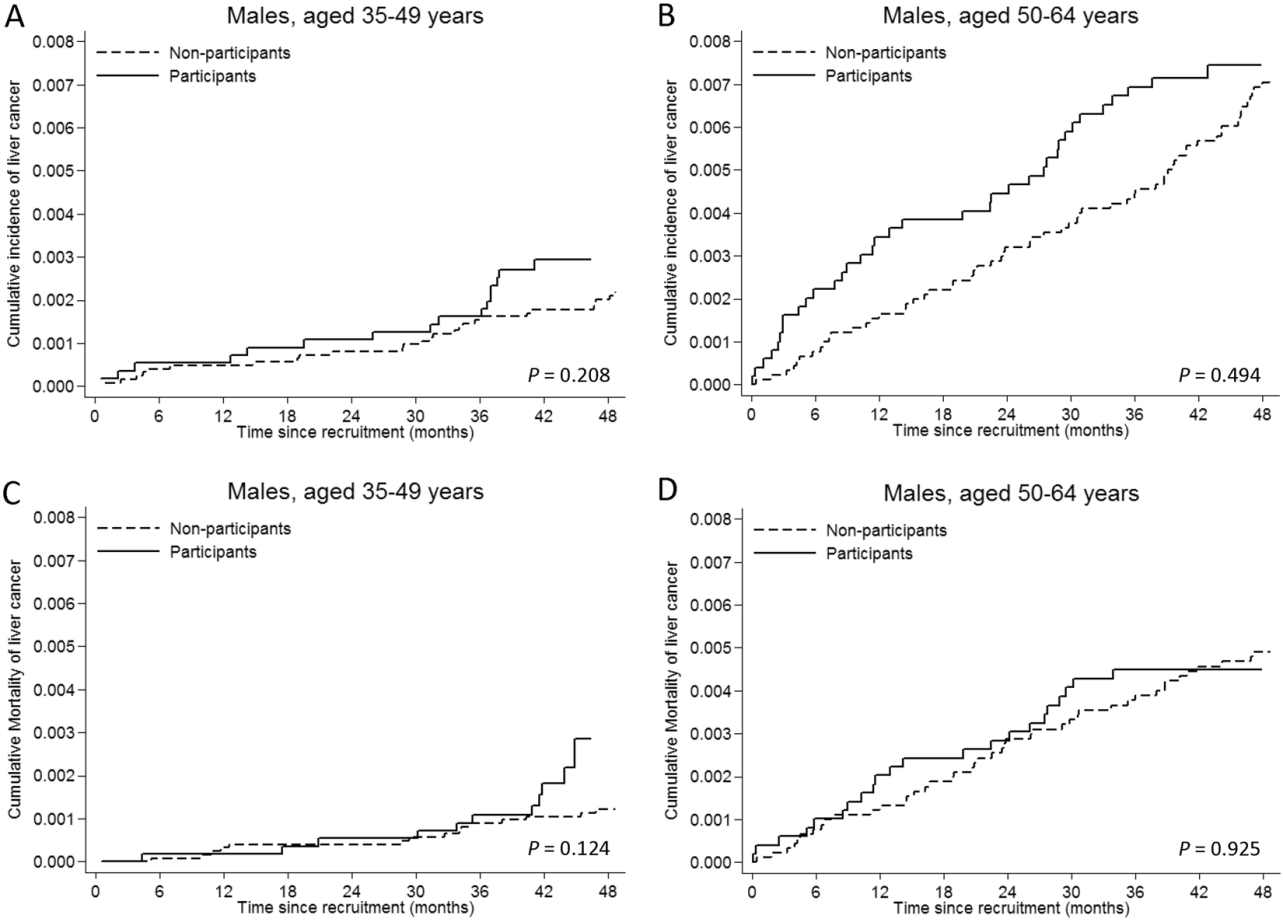
Cumulative incidence and mortality of liver cancer according screening status in males stratified by age group at study entry. Poisson regression models were used to calculate *P* values.

From the Death Register, 29 deaths had been recorded due to liver cancer but had not been recorded in the Cancer Register (due to a delayed report). Among them, 9 cases were non-liver cancer metastatic to the liver thus were excluded as liver cancer cases but were censored at their primary cancer diagnosis. Table 3 shows liver cancer incidence and mortality among participants and non-participants. Within 4 years of follow-up, we identified 57 incident liver cancer cases among participants (incidence rate 79.4 per 100,000 person-years, standardized to age distribution of person-years experienced by all residents using 5-year age categories). Compared with non-participants (104 incident cases of liver cancer; age-adjusted incidence rate 54.6 per 100,000 person-years), liver cancer incidence among participants was slightly increased, albeit not significantly (age- and sex-adjusted RR = 1.21, 95% CI = 0.88, 1.68, *P* = 0.244). Specifically, liver cancer incidence increased in 2012 (the initial year of screening, *P* = 0.042), but was not increased in the subsequent years. Stratified analyses showed that liver cancer incidence did not differ significantly between participants and non-participants in different sex and age groups (Table 3; Fig. 2A,B for males, and Fig. S1A,B for females).

**Table 3:** Liver cancer incidence and mortality 2012–2015*.

	Participants (N = 17,966)		Non-participants (N = 50,544)		Participants vs. non- participants	
	No. of cases	Rate per 100,000 person-years^†^ (95% CI)	No. of cases	Rate per 100,000 person-years^†^ (95% CI)	Adjusted relative risk (95% CI)^††^	*P* value
**Liver cancer incidence**	57	79.4	104	54.6	1.21 (0.88, 1.68)	0.244
**Sex**						
Male	52	134.2	91	109.5	1.21 (0.86, 1.70)	0.275
Female	5	13.1	13	12.3	1.13 (0.40, 3.20)	0.813
**Age in 2012 (years)**						
35–49	17	57.2	30	25.0	1.46 (0.80, 2.66)	0.215
50–64	30	86.5	74	92.0	1.13 (0.77, 1.66)	0.530
**Calendar year**						
2012	15	115.0	25	55.2	1.95 (1.02, 3.71)	0.042
2013	11	55.6	22	45.1	1.03 (0.50, 2.14)	0.931
2014	16	73.1	24	49.8	1.36 (0.72, 2.57)	0.346
2015	15	80.9	33	61.6	0.89 (0.48, 1.65)	0.721
**All-cause mortality**	274	376.2	699	369.7	0.91 (0.79, 1.04)	0.167
**Liver cancer mortality**	33	44.9	69	36.2	1.04 (0.68, 1.58)	0.856
**Sex**						
Male	33	84.7	59	70.9	1.18 (0.77, 1.80)	0.456
Female	0	0.0	10	9.4	0	N/A
**Age in 2012 (years)**						
35–49	11	37.0	17	14.1	1.66 (0.77, 3.56)	0.193
50–64	22	63.3	52	64.6	0.88 (0.53, 1.45)	0.611

*The median follow-up time for incidence was 4.0 years (interquartile range, 3.8 to 4.0), and for mortality was 4.0 years (interquartile range, 3.8 to 4.0).

^†^Incidence rate per 100 000 person-years, standardized to age distribution of person-years experienced by all residents using 5-year age categories.

^††^Adjusted for sex and age. For analyses stratified by sex, models were only adjusted for age. For analyses stratified by age, models were only adjusted for sex.

Liver cancer cases detected among participants had better prognosis than those detected among non-participants (sex- and age- adjusted HR = 0.64, 95% CI = 0.42, 0.98; Fig. S2). Multivariable models further adjusting for clinical stage, treatment, serum alanine transaminase (ALT) levels, albumin, bilirubin, and prothrombin showed that HR was attenuated (HR = 0.78, 95% CI = 0.48, 1.27). The 1-year and 3-year overall survival proportions for liver cancer cases identified among participants were 48.7% (95% CI = 34.3%, 61.6%) and 29.1% (95% CI = 15.4%, 44.3%), respectively; for those identified among non-participants, the 1- and 3-year overall survival proportions were 36.9% (95% CI = 26.6%, 47.1%) and 15.5% (95% CI = 7.7%, 25.7%), respectively.

However, all-cause mortality was similar among participants and non-participants (age- and sex-adjusted RR = 0.91, 95% CI = 0.79, 1.04, *P* = 0.167). Furthermore, liver-cancer-specific mortality did not differ significantly overall (RR = 1.04, 95% CI: 0.68, 1.58, P = 0.856) or by age group (Table 3). Because all 5 female liver cancer cases detected among participants were still alive at the end of follow-up, we could not estimate the RR for liver cancer-specific mortality among females. Nevertheless, analysis limited to males showed no significant differences between participants and non-participants (Fig. 2C,D).

Among 57 cases of liver cancer detected among participants during 4 years of follow-up, 35.1% (N = 20) were detected within one year after study entry (Table 4). Liver cancer cases among participants and non-participants were similar in terms of age and sex, but those among study participants were slightly more likely to be diagnosed at early stages (Barcelona Clinic Liver Cancer [BCLC] stage 0 or A; 22.8% vs 14.4%). Of 13 early-stage cases detected among participants, treatment information was missing for two cases. Nine cases (69.2%) underwent surgical resection, and two cases (15.4%) underwent locally ablative therapy. Of 15 early-stage cases detected among non-participants, nine cases (60.0%) underwent surgical resection and 5 cases (40.0%) underwent locally ablative therapy.

**Table 4:** Liver cancer incidence and stage according to study participation, 2012–2015.

Variable	Participants			Non-participants	*P* value^†^
	No. at baseline*	No. at follow-up	Total no. (%)	Total no. (%)	
Total	20	37	57 (100.0)	104 (100.0)	
Sex					0.604
Male	20	32	52 (91.2)	91 (87.5)	
Female	0	5	5 (8.8)	13 (12.5)	
Age in 2012 (years)					0.400
35–39	1	0	1 (1.8)	8 (7.7)	
40–49	2	14	16 (28.1)	22 (21.2)	
50–59	14	14	28 (49.1)	52 (50.0)	
60–64	3	9	12 (21.0)	22 (21.1)	
First-degree family history of cancer					
No	20	35	55 (96.5)	—	
Cancers other than liver	0	0	0 (0.0)	—	
Liver cancer	0	2	2 (3.5)	—	
Diagnosis method					0.978
CT or MRI	9	27	36 (63.2)	64 (61.5)	
Pathology	11	8	19 (33.3)	36 (34.6)	
Unknown	0	2	2 (3.5)	4 (3.9)	
Clinical stage (BCLC)					0.466
0 or A (Very early or early stage)	6	7	13 (22.8)	15 (14.4)	
B (Intermediate stage)	6	9	15 (26.3)	25 (24.0)	
C (Advanced stage)	6	13	19 (33.3)	45 (43.3)	
D (Terminal stage)	1	6	7 (12.3)	14 (13.5)	
Unknown	1	2	3 (5.3)	5 (4.8)	

Abbreviations: BCLC, Barcelona Clinic Liver Cancer; CT, computerized tomography; MRI, magnetic resonance imaging.

*Defined as cases of liver cancer identified within one year after study entry.

^†^*P* value from chi-squared test comparing study participants and non-participants.

Finally, among 43 liver cancer cases detected from the group positive for HBsAg, we identified 9 patients who did not attend any ultrasonography during follow-up, 10 patients who attended ultrasonography screening but at a lower frequency than recommended, and 24 patients who attended ultrasonography screening at the recommended frequency (every 6 months). Liver cancer incidence and mortality by surveillance compliance status are provided in Supplementary Table 2. In addition, patients who attended ultrasonography screening at the recommended frequency had higher HCC incidence but a better prognosis than those who did not attend ultrasonography screening at the recommended frequency or at all during follow-up, although the difference in prognosis was not statistically significant (adjusted HR for overall survival = 0.42, 95% CI = 0.16, 1.11, *P* = 0.080).

### Incidence and stage of liver cancer according to detection methods

We identified 14 cases of liver cancer among participants who were HBsAg-seronegative at baseline, of whom 3 were positive for anti-HCV antibodies; 3 of these were diagnosed at an early stage (21.4% of 14, Table 5). This early detection rate is similar to that among liver cancer cases identified among participants who were HBsAg seropositive at baseline (10 cases, 23.2% of 43). Of the 43 HBsAg-positive patients, only 8 (18.6%) had elevated AFP at baseline, although 39.5% (17 of 43) had a positive AFP result at baseline or during follow-up. The early detection rate of 23.5% (4 of 17) among HBsAg-positive liver cancer patients who had elevated AFP is similar to the rate of 23.1% (6 of 26) among HBsAg-positive patients who were AFP negative. Among 25 liver cancer cases who underwent baseline ultrasonography, 76.0% (19 of 25) had an abnormal ultrasonography result; of these, 15.8% (3 of 19) were diagnosed at an early stage. Liver cancer cases who underwent at least one ultrasonography appeared to have better overall survival during follow-up than those who did not (case fatality rate 45.2% vs 66.7%).

**Table 5:** Liver cancer stage according to serological results and liver ultrasonography, 2012–2015.

	Cancer stage (BCLC)					Total
	0 or A	B	C	D	missing	
**HBsAg status at baseline**						
Negative						
Cases of liver cancer (no.)	3	2	5	3	1	14
Deaths from liver cancer (no. %)	1 (33.3)	2 (100.0)	5 (100.0)	3 (100.0)	0 (0.0)	11 (84.6)
Positive						
Cases of liver cancer (no.)	10	13	14	4	2	43
Deaths from liver cancer (no. %)	2 (20.0)	7 (53.8)	11 (78.6)	2 (50.0)	0 (0.0)	22 (51.2)
**Among HBsAg-positive group only**						
**AFP (baseline)**						
Negative						
Cases of liver cancer (no.)	7	11	11	4	2	35
Deaths from liver cancer (no. %)	1 (14.3)	6 (54.5)	8 (72.7)	2 (50.0)	0 (0.0)	17 (48.6)
Positive						
Cases of liver cancer (no.)	3	2	3	0	0	8
Deaths from liver cancer (no. %)	1 (33.3)	1 (50.0)	3 (100.0)	0 (0.0)	0 (0.0)	5 (62.5)
**AFP (baseline + follow-up)**						
Negative						
Cases of liver cancer (no.)	6	8	8	2	2	26
Deaths from liver cancer (no. %)	1 (16.7)	3 (37.5)	6 (75.0)	1 (50.0)	0 (0.0)	11 (42.3)
Positive						
Cases of liver cancer (no.)	4	5	6	2	0	17
Deaths from liver cancer (no. %)	1 (25.0)	4 (80.0)	5 (83.3)	1 (50.0)	0 (0.0)	11 (64.7)
**Ultrasonography (baseline)**						
Normal						
Cases of liver cancer (no.)	1	0	4	0	1	6
Deaths from liver cancer (no. %)	0 (0.0)	0 (0.0)	3 (75.0)	0 (0.0)	0 (0.0)	3 (50.0)
Abnormal						
Cases of liver cancer (no.)	3	6	8	2	0	19
Deaths from liver cancer (no. %)	0 (0.0)	3 (50.0)	7 (87.5)	1 (50.0)	0 (0.0)	11 (57.9)
Missing						
Cases of liver cancer (no.)	6	7	2	2	1	18
Deaths from liver cancer (no. %)	2 (33.3)	4 (57.1)	1 (50.0)	1 (50.0)	0 (0.0)	8 (44.4)
**Ultrasonography (baseline + follow-up)**						
Normal						
Cases of liver cancer (no.)	0	0	0	0	0	0
Deaths from liver cancer (no. %)	0 (0.0)	0 (0.0)	0 (0.0)	0 (0.0)	0 (0.0)	0 (0.0)
Abnormal (any)						
Cases of liver cancer (no.)	6	9	12	3	1	31
Deaths from liver cancer (no. %)	0 (0.0)	4 (44.4)	9 (75.0)	1 (33.3)	0 (0.0)	14 (45.2)
Missing (all)						
Cases of liver cancer (no.)	4	4	2	1	1	12
Deaths from liver cancer (no. %)	2 (50.0)	3 (75.0)	2 (100.0)	1 (100.0)	0 (0.0)	8 (66.7)

Abbreviations: AFP, alpha-fetoprotein; BCLC, Barcelona Clinic Liver Cancer; HBsAg, Hepatitis B virus surface antigen.

### Quality of liver cancer incidence and mortality ascertainment

To estimate the completeness of ascertainment of liver cancer incidence and mortality through the demonstration project, and to test whether the target community of Xiaolan Town was representative of the population of Zhongshan City, we compared liver cancer incidence, disease-specific mortality, and all-cause mortality rates between the study population and the general population of Zhongshan City in 2012 and 2013. We observed no significant differences in the three measures. The SIR for liver cancer was 1.14 (95% CI = 0.97, 1.32, *P* = 0.119); the SMR for liver cancer was 1.05 (95% CI = 0.88, 1.25, *P* = 0.563); and the SMR for all causes was 1.06 (95% CI = 0.99, 1.13, *P* = 0.076).

Of 500 subjects randomly selected from those who participated a screening trial for nasopharyngeal carcinoma but not for liver cancer, 496 subjects were tested for HBsAg, of whom 15.5% (N = 77) were positive. The positive rate did not differ from that of participants in the screening for liver cancer (*P* = 0.844). Of 77 subjects who were positive for HBsAg, only 4 had ever attended AFP and/or ultrasonography examination and undergone anti-viral treatment.

Sensitivity analysis by starting follow-up one year after the date of enrollment showed results comparable to those of the main analyses. For example, liver cancer-specific mortality among participants did not differ significantly to that among non-participants (RR = 0.99, 95% CI = 0.59, 1.65).

## Discussion

In the first 4 years of follow-up, we found suggestive evidence that the current screening strategy for liver cancer recommended in China can detect more cases (especially in the initial year of screening, and perhaps for early-stage cases). It also appeared that survival was better for screen-detected liver cancer cases than for cases among non-participants. However, the lack of a short-term benefit on reduction of mortality due to liver cancer indicates that this pattern might best be explained by lead time bias, i.e., that prevalent liver cancers are detected slightly earlier by screening without a true impact on the disease course. Moreover, the recommended screening strategy may have poor acceptability and compliance in the general population.

There are several potential explanations for the findings. First, AFP has poor sensitivity as a liver cancer screening biomarker, especially for early-stage disease, and thus may not be useful for mass screening^18^. The reported sensitivity of ultrasonography imaging in detecting tumor nodules has been quite variable, ranging from 30–100%^19^, depending on the expertise of the operator as well as the technological sophistication of the equipment. In Zhongshan City, where the economy is still developing, we lacked both advanced imaging equipment and highly experienced radiologists. Thus, our results may point to a need for less labor-intensive or technology-dependent screening methods that can be implemented in less economically developed areas. Second, the compliance of HBsAg-positive subjects in subsequent liver cancer screening was not high, with 61.8% attending the first round of scheduled liver ultrasonography, and fewer participating in subsequent ultrasonography. If participation in liver ultrasonography had been greater, the early detection rate may have been better among the 12 liver cancer cases ultimately observed among HBsAg-positive subjects who skipped liver ultrasonography. However, the distribution of clinical stage was similar between cases detected from subjects who never participated any AFP/liver ultrasonography and those who participated in at least one AFP/liver ultrasonography (*P* = 0.672). Third, compliance with the recommendation of undergoing ultrasonography every 6 months was low, with only 7.3% of HBsAg-positive participants completing the recommended schedule of ultrasonography. Patients who followed the recommended screening had a better prognosis than those who did not. This evidence underscores the importance of following recommended guidelines when conducting screening for liver cancer, and maximizing compliance through patient education and communication. In addition, measures of motivating participation of ultrasonography should be considered, including repeated reminding through various approaches, paying visits to the patients’ home, and compensation for personal leave due to attending ultrasonography examination, etc. Fourth, non-participants resided in the same community as participants, making them susceptible to influence by the screening program. Increased awareness of liver cancer risk and screening may have encouraged non-participants to seek clinical examination independently of the study.

The non-significantly increased incidence of liver cancer among participants is less likely to be confounded by unmeasured factors because the seroprevalence of HBsAg among patients with liver cancer is identical to other reports in China^5^. Only 5% (4 of 77) of non-participants who were HBsAg-positive underwent AFP/ultrasonography examination, indicating that our results are unlikely to be confounded by liver surveillance among non-participants, assuming the randomly selected subjects were representative of the general population. Our study personnel, including local health care providers throughout the study area, are not aware of any other large-scale liver cancer screening programs in the region. The RR of liver-cancer specific mortality for screening was close to 1.0, which raises a debate on whether increasing sample size could increase the power to detect a significant reduction of liver-cancer- specific mortality. The mass screening strategy currently recommended in China largely relies on the HBsAg test only. This approach overlooks ~30% of liver cancer in China not attributable to HBV (e.g. due to infection with hepatitis C virus [HCV], exposure to aflatoxin, alcohol drinking, or smoking)^4,5^. For example, in the present study, 24% (13 of 54) of liver cancer cases detected among participants were HBsAg-negative at baseline, and 84.6% of these patients died from liver cancer, compared with 51.2% of HBsAg-positive patients. This result underscores the need to improve the current screening strategy to cover causes other than HBV infection. For example, a cohort study in Taiwan showed that use of transaminases (ALT and aspartate transaminase [AST]) regardless of HBV or HCV status can achieve a high accuracy of HCC risk prediction^20^. An implementation of a risk-score-guided screening study in Taiwan led to a significant reduction in liver cancer mortality in the general population^21^. At the same time, laboratory-based development of more accurate liver cancer biomarkers is needed to improve early diagnosis.

Previous studies usually focused on high-risk populations, for example, only HBsAg-positive individuals^14,22,23^; thus, their results may not apply to general populations in a community setting. To the best of our knowledge, the present study is the first to report both liver cancer incidence and liver-cancer-specific mortality rates in a mass screening study in China. Other strengths of our study include medical record review and diagnostic confirmation of all liver cancer cases by one gastroenterologist. Although it would be difficult to confirm the true underlying causes of death among patients of liver cancer, liver cancer is a highly lethal malignancy with 5-year survival rate of <5%, such that misclassification should be minimal due to a short period of follow-up in our screening trial. Although follow-up time is systematically different between the participant and non-participant groups, we used person-years, which largely reduced such difference. However, our results should be interpreted in light of a few methodological limitations. First, our study had a relatively short follow-up; therefore, long-term efficacy of the recommended screening strategy is uncertain. Second, although our study was conducted in an area with relatively good health care facilities, gastroenterological specialist services remain unevenly distributed. Because evaluation of imaging from ultrasonography depends on the experience of specialists, the relatively low proportion of early-stage liver cancer cases in our study, as compared with other screening trials, may indicate the need to improve training of local gastroenterologists. Third, because our study was not a randomized controlled trial, baseline characteristics were unevenly distributed, raising concerns of confounding. We adjusted for differences in sex and age between the two groups, but imbalances in other unmeasured risk factors may have contributed to an underestimate of screening efficacy. The low prevalence of a family history of liver cancer and HCV infection in the study area limits the potential impact of confounding by these risk factors. Fourth, compliance with semi-annual ultrasonography was poor, and reasons for this noncompliance (e.g., insufficient education of participants about the importance of regular screening, or inadequate communication to remind participants of scheduled ultrasonography appointments) should be investigated and ameliorated. Finally, non-participants were followed up only through linkage to the cancer register, probably leading to under ascertainment of HCC cases, particularly for early-stage disease. However, missed HCC cases would not affect the estimate of HCC-specific mortality.

## Conclusions

In conclusion, our demonstration screening study does not show a reduction in liver cancer mortality after 4 years based on the currently recommended screening strategy for liver cancer in China, which defines high-risk individuals based on HBsAg positivity alone. In addition, the incidence of liver cancer among participants was 0.08% per year, which does not meet the threshold (i.e., 0.2% per year for HBV carriers) for beneficial and cost-effective cancer surveillance according to AASLD guidelines^24^. These results may lend support to a more targeted screening strategy, as currently recommended by AASLD and APASL^10,11^, rather than a community-based mass-screening approach. Although compliance with the recommended screening strategy was low, continued screening will be conducted among HBV carriers, thus long-term efficacy remains to be evaluated in this study population. Improvement of compliance with cancer screening guidelines among participants, and development of risk score-guided screening designed specifically for the Chinese population, along with identification of more accurate biomarkers for early diagnosis, may enable more effective mass screening.

## Electronic supplementary material

Supplementary figures and tables
