# Supplementary material for: Mass screening for liver cancer: results from a demonstration screening project in Zhongshan City, China
Source: Sci Rep. 2018 Aug 24;8:12787. doi: 10.1038/s41598-018-31119-9 (PMC6109066; doi:10.1038/s41598-018-31119-9)
Supplement: Supplementary file 1 — Supplementary figures and tables [file 41598_2018_31119_MOESM1_ESM.pdf]

Mass screening for liver cancer: results from a demonstration screening project in Zhongshan City, China

Ming-Fang Ji [1 †], Zhiwei Liu [2,3 †], Ellen T. Chang [4,5], Xia Yu [1], Biao-Hua Wu [1], Li Deng [1], Qian-Jin Feng [1], Kuang-Rong Wei [1], Xue-Jun Liang [1], Shi-Feng Lian [1], Wen Quan [1], Pan-Pan Wang [1], Yun Du [1], Zhi-Heng Liang [1], Sheng-Lin Xia [6], Hai Lin [6], Fu-Gui Li [1], Wei-Min Cheng [1], Wei-Qiang Chen [1], Yong Yuan [1,#], and Weimin Ye [2,7,#,*]

Authors' affiliations

1. Cancer Research Institute of Zhongshan, Zhongshan City People's Hospital, Zhongshan, Guangdong, P. R. China
2. Department of Medical Epidemiology and Biostatistics, Karolinska Institutet, Stockholm, Sweden
3. Division of Cancer Epidemiology and Genetics, National Cancer Institute, 9609 Medical Center Drive, Rockville, Maryland, USA
4. Exponent, Inc., Health Sciences, Menlo Park, California, USA
5. Division of Epidemiology, Department of Health Research and Policy, Stanford University School of Medicine, Stanford, California, USA
6. Institute for Prevention & Control of Non-Communicable Chronic Diseases, Zhongshan Center for Disease Control and Prevention, Zhongshan, Guangdong, P. R. China
7. Sun Yat-sen University Cancer Center, State Key Laboratory of Oncology in South China, Collaborative Innovation Center for Cancer Medicine, Guangzhou, P. R. China

† Authors contributed equally.

# Shared senior authors.

Corresponding author

Weimin Ye, Sun Yat-sen University Cancer Center, State Key Laboratory of Oncology in South China, Collaborative Innovation Center for Cancer Medicine, Guangzhou 510060, P. R. China. Phone: 86-20-87345685, Fax: 86-20-87343392, Email: yewm@sysucc.org.cn

Number of figures and tables: 6

Competing interests: None declared.

Grant Support: This work was funded by grants from the National Natural Science Foundation of China (Grant # 81672098), Central Financial Transfer Payment Projects of the Chinese Government, Cancer Research Grant of Zhongshan City, and Key Programs of Medical Research of Zhongshan City People's Hospital. WY was supported in part by grants from Sun Yat-sen University and Sun Yat-sen University Cancer Center.

List of Abbreviations: AFP, alpha-fetoprotein; BCLC, Barcelona Clinic Liver Cancer; CT, computerized tomography; HBsAg Hepatitis B virus surface antigen; HBV, Hepatitis B virus; HCV, Hepatitis C virus; MRI, magnetic resonance imaging; SIR, standardized incidence rate; SMR, standardized mortality rate

Contributors: Study conception and design: MFJ, ZL, YY, WY. Acquisition of data: MFJ, XY, BHW, LD, QJF, KRW, XJL, SFL, WQ, PPW, YD, ZHL, SLX, HL, FGL, WMC, WQC, YY. Statistical analysis: ZL. Analysis and interpretation of data: MFJ, ZL, ETC, WY. Drafting of manuscript: MFJ, ZL, ETC, WY. Critical revision: ETC, YY, WY.

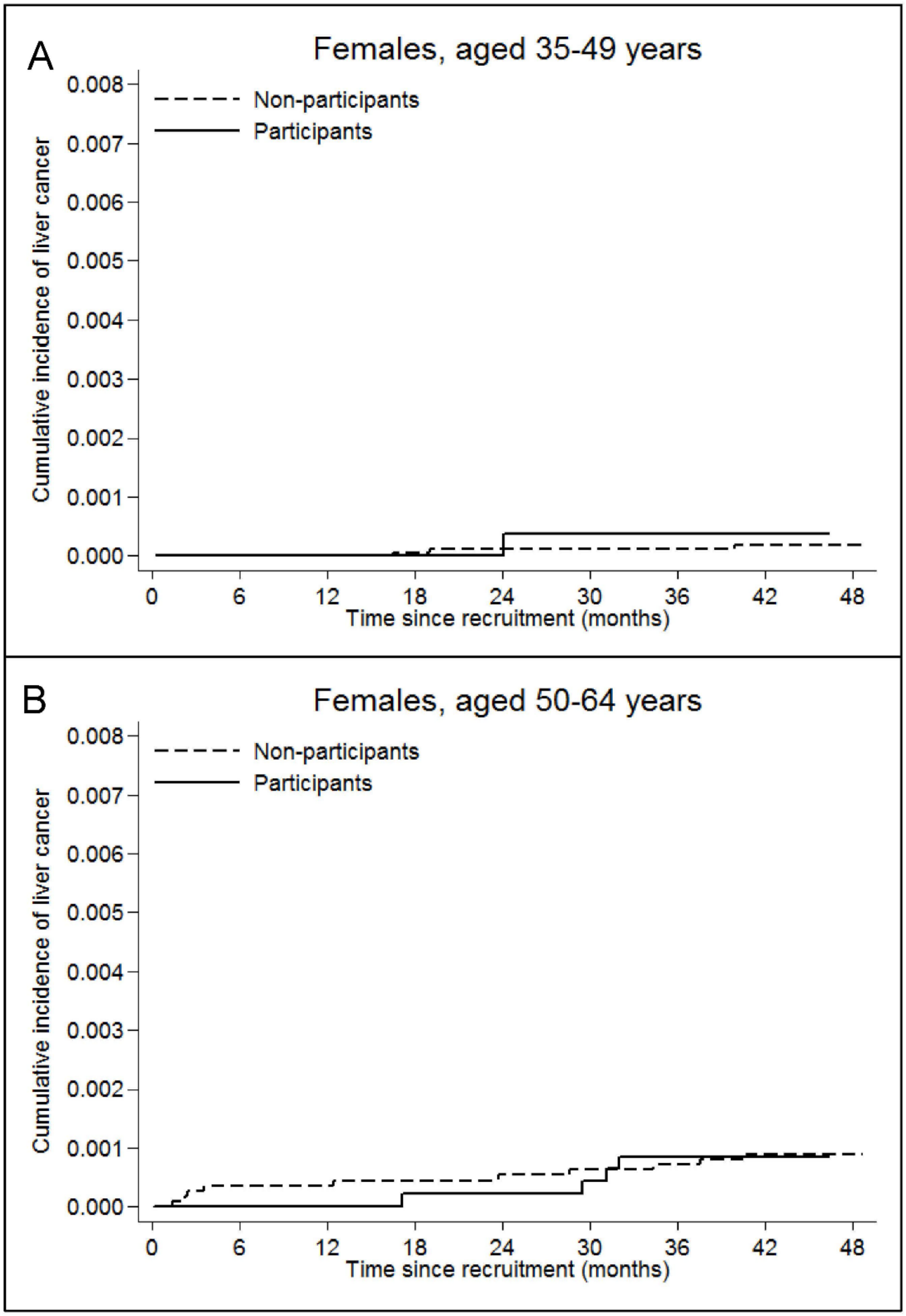

Figure S1. Cumulative incidence of liver cancer according screening status in females stratified by age group at study entry.

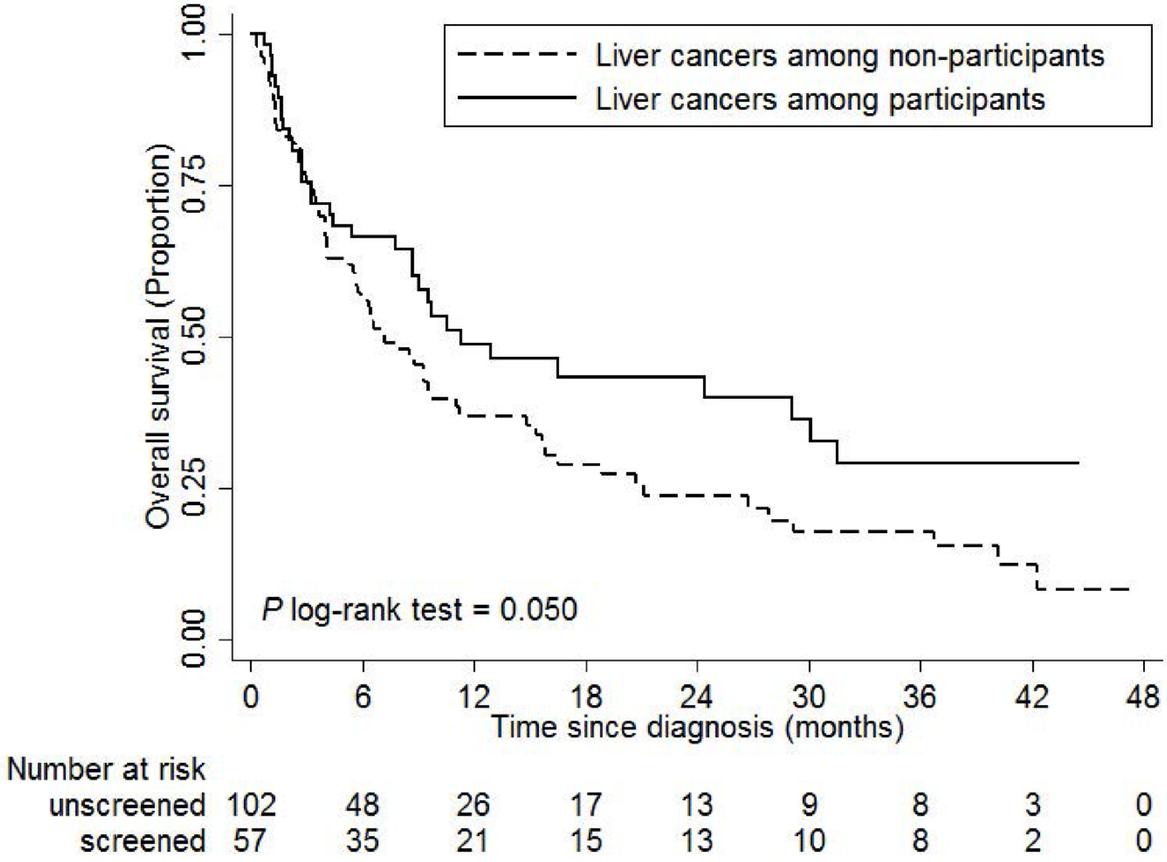

Figure S2. Kaplan-Meier survival estimates for patients with liver cancer in participation group and non-participation group. Log-rank test was used to calculate *P* values. Two cases were excluded due to no follow-up time.

Supplementary Table 1. Number of participants in re-examination during follow-up among subjects positive for hepatitis B surface antigen (N=2848), 2012-2015. *

	Gender		Age in 2012 (years)			
	**male**	**female**	**35-39**	**40-49**	**50-59**	**60-64**
**AFP**						
Baseline†	1914	934	421	1173	974	280
1st round	696	438	131	436	424	143
2nd round	1092	507	212	619	597	171
3rd round	922	402	168	540	484	132
4th round	1190	592	225	688	672	197
5th round	983	504	189	583	564	151
**Ultrasonography**						
Baseline	1167	593	237	693	644	186
1st round	402	207	94	257	206	52
2nd round	1031	482	203	611	545	154
3rd round	936	412	176	562	490	120
4th round	1032	507	203	584	584	168
5th round	911	491	170	527	548	157

* Dates for the first to the fifth round of re-examinations: October 2012 - August 2013, April 2014, October 2014, April 2015, and October 2015, respectively.

† Baseline serum samples were used for testing for AFP thus all subjects had baseline AFP information.

Abbreviation: alpha-fetoprotein, AFP

Supplementary Table 2. Liver cancer incidence and mortality by surveillance compliance status, 2012-2015 *

Participant	HBsAg	Ultrasonography examinations	Total No.	No. of liver cancer	Incidence RR (95% CI)	Cancer-specific mortality RR (95% CI)	Overall mortality RR (95% CI)
No	Unknown	No	50,544	104	ref.	ref.	ref.
Yes	-	No	15,118	14	0.35 (0.20, 0.61)	0.41 (0.21, 0.77)	0.88 (0.76, 1.02)
	+	Not as recommended	2,641	19	2.82 (1.73, 4.61)	2.88 (1.59, 5.22)	0.90 (0.65, 1.26)
	+	As recommended	207	24	47.9 (30.7, 74.8)	23.9 (11.9, 47.8)	2.68 (1.39, 5.17)

* Relative risk (RR) was adjusted for sex and age.
